# Carbonatogenic Bacteria in the Maros-Pangkep Karst: Protectors or Threat to Prehistoric Paintings?

**DOI:** 10.4014/jmb.2410.10019

**Published:** 2025-02-20

**Authors:** Nur Haedar, Faisal, Nur Afifah Zhafirah, Riuh Wardhani, Asadi Abdullah, Rustan Lebe, Fuad Gani, Heriadi

**Affiliations:** 1 Department of Biology, Faculty of Mathematics and Natural Sciences, Universitas Hasanuddin, Makassar, South Sulawesi 90245, Indonesia; 2 Division of Animal and Dairy Science, Chungnam National University, Daejeon 34134, Republic of Korea; 3 Cultural Heritage Conservation Center, Makassar 90245, Indonesia

**Keywords:** Carbonatogenic bacteria, CaCO_3_ precipitation, cultural heritage, Maros-Pangkep karst, CaCO_3_

## Abstract

The Maros-Pangkep karst region hosts prehistoric cave paintings recognized by UNESCO as a world cultural heritage site. The presence of calcium carbonate (CaCO_3_) on the surface of these artworks suggests the involvement of carbonatogenic bacteria, which facilitate CaCO_3_ production or deposition. While these bacteria have been explored for their potential in stone artwork conservation, their role in either preserving or obscuring prehistoric paintings remains unclear. This study aims to identify carbonatogenic bacteria associated with the Maros-Pangkep cave paintings and evaluate their CaCO_3_ precipitation potential. Bacteria were isolated using Calcium Carbonate Precipitation (CCP) medium, and their CaCO_3_ precipitation capacity was assessed by measuring precipitate mass and ammonia (NH_3_) levels. Molecular identification was conducted using 16S rRNA gene sequencing. Eighteen bacterial isolates were obtained from swab samples collected in Parewe and Bulu Sipong caves, ten of which were identified as carbonatogenic. Among these, two isolates exhibited the highest CaCO_3_ precipitation: Ps1-d produced 2.45 ± 0.07 mg/ml CaCO_3_ with 946.3 ± 26.3 mg/l NH_3_, while Ps8-b produced 1.80 ± 0.05 mg/ml CaCO_3_ with 763.4 ± 21.2 mg/l NH_3_. Molecular analysis identified Ps1-d as *Bacillus* cereus strain bk and Ps8-b as *Bacillus* sp. NCCP-428. These findings have significant implications for both (1) the potential application of carbonatogenic bacteria in the conservation and restoration of stone artworks and (2) the development of strategies to inhibit excessive CaCO_3_ deposition to prevent the obscuration of cultural heritage paintings.

## Introduction

Karst landscapes are regions where erosion caused by the dissolution of rock leads to the creation of caves, sinkholes, and large springs. These landscapes and their underlying aquifer systems cover roughly 15% of the Earth's ice-free land surface, making them crucial as water resources since they provide drinking water to an estimated 13-25% of the global population [1]. These regions are characterized by distinctive features, including rocky terrain, caves, underground rivers, and unusual geological formations [2-7]. In addition, karst landscapes can also develop in non-carbonate rocks, such as quartzite and silica sandstone [8]. One notable karst region recognized by UNESCO as a World Heritage Site is the Maros-Pangkep Karst. This karst system is renowned for its spectacular tower formations and contains 572 caves, including one that extends 27 km and connects an underground river to dolines. Over 332 prehistoric artifacts have been discovered in the region, including the world’s oldest known figurative artwork, a 45,500-year-old depiction of a pig found on a wall in Leang Tedongnge cave.

Archaeological studies have uncovered evidence of human use of caves since the Paleolithic period [9]. These caves functioned not only as shelters and storage spaces but also as sites for artistic expression, burials, and ceremonies [10, 11]. Many karst areas are designated as UNESCO World Heritage Sites due to their historical and cultural importance. The prehistoric paintings on cave walls in these regions are but one fascinating aspect of this cultural heritage. Such paintings provide insights into the experiences, struggles, and aspirations of prehistoric humans. However, over time, many of these paintings have suffered damage and are at risk of extinction [12-14]

Prehistoric paintings in karst caves are subject to damage by natural processes and human activities, including physical and chemical weathering [15, 16]. Mualliful *et al*. (2024) identified that physicochemical processes play a significant role in the darkening of rock art in the Maros-Pangkep Karst [17]. Their SEM-EDS analysis revealed the presence of gypsum with filament-like structures, which were especially prominent in the more heavily darkened samples, suggesting that both geochemical and microbial factors contribute to the formation of these deposits [17] Consequently, it is crucial to further investigate the relationship between the degradation of prehistoric paintings on the walls of karst caves and microbial activity, particularly the presence of carbonatogenic bacteria [6, 18, 19]. Studies have identified various types of microorganisms in karst regions, with Proteobacteria, *Actinomycetes*, and Firmicutes being the dominant communities present [20, 21]. Notably, carbonatogenic bacteria have been found to precipitate carbonate on karst rock surfaces. However, studies specifically investigating the specific microbes that play roles on the deterioration of prehistoric paintings in the Maros-Pangkep karst region remain limited.

This study focuses on the isolation, characterization, and identification of carbonatogenic bacteria responsible for the deterioration of cave wall paintings in the Maros-Pangkep karst region. These bacteria produce CaCO_3_, which forms a layer over the paintings, leading to their degradation. The production of CaCO_3_ is driven by Microbially Induced Calcite Precipitation (MICP), a natural process in which microorganisms alter the geochemistry of their surrounding environment [22, 23]. MICP occurs through bacterial metabolic pathways that promote carbonatogenesis. The ureolytic metabolic pathway is the most common mechanism for CaCO_3_ precipitation in bioconsolidation, where the formation of carbonate, NH_3_, and hydroxyl ions increases pH in an alkaline environment [24-26]. Here, carbonatogenic bacteria are identified based on their ability to precipitate CaCO_3_ and produce NH_3_ as an intermediate in the MICP process. Additionally, promising bacterial strains were further identified through molecular analysis.

## Materials and Methods

### Study Area

**Fig. 1:**
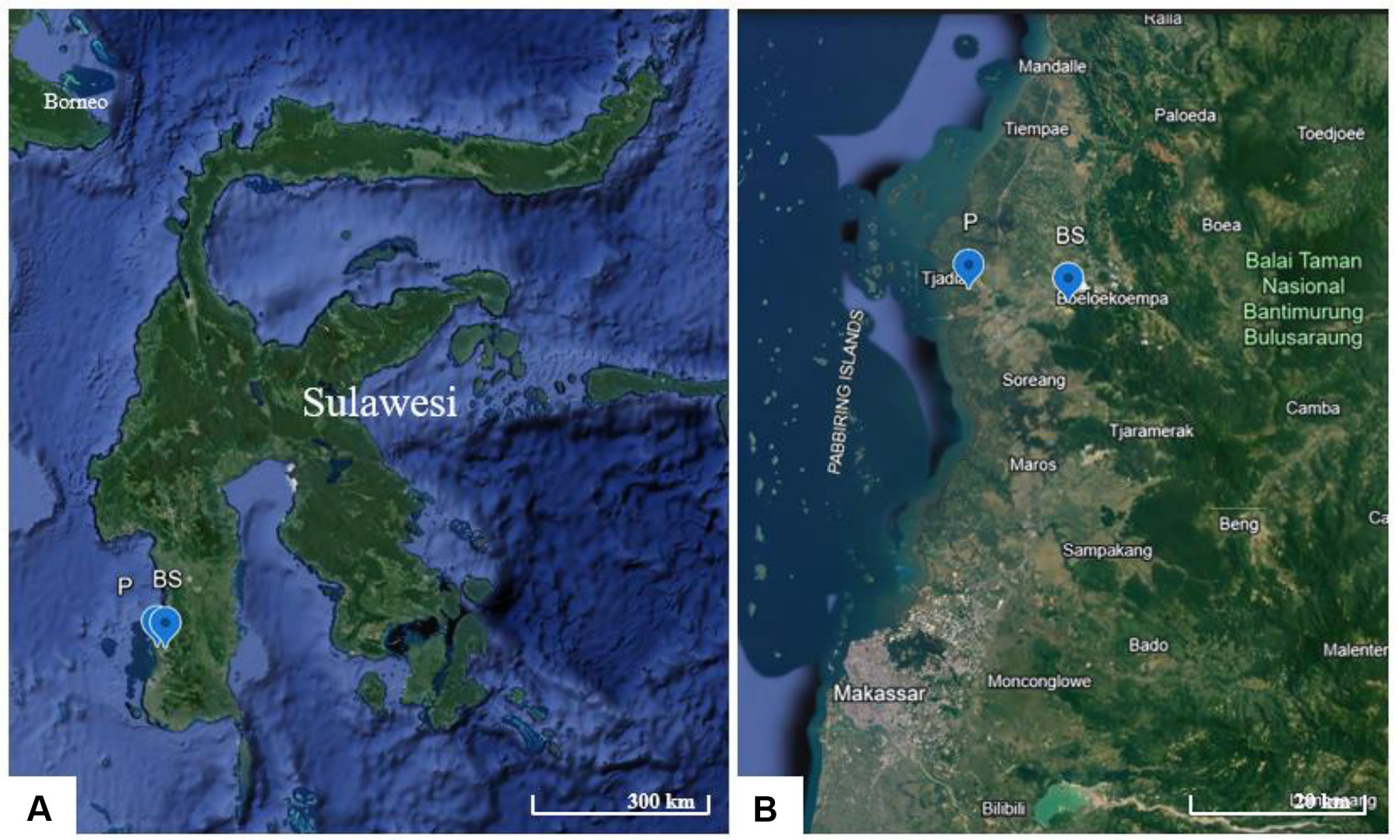
Sampling sites of carbonatogenic bacteria in Maros-Pangkep karst area; (P) Parewe cave; (BS) Bulu Sipong 4 cave.

The sampling sites for the carbonogenic bacterial isolates responsible for the prehistoric painting damage in this study are in the Pangkajene and Islands Regency, specifically at two key locations: Leang Parewe and Leang Bulu Sipong 4. Leang Parewe is in Borimasunggu, Labakkang Subdistrict, at coordinates 4°47'40.1''S, 119°31'7.25''E. Leang Bulu Sipong 4 is in Bontoa, Minasatene Subdistrict, at coordinates 4°48'23.81''S, 119°36'36.9''E (Fig. 1A and 1B). Both cave sites are rarely visited, meaning minimal human disturbance has led to well-preserved and relatively pristine conditions.

An examination of the diversity of the prehistoric paintings at the two sites revealed that Leang Parewe contains only handprint paintings, while Leang Bulu Sipong 4 features a wider variety, including handprints, footprints, human figures, dogs, anoa (dwarf buffalo), and babirusa (deer-pigs). The oldest images are found on a 4.5-m limestone rock art panel in Leang Bulu Sipong 4, which depicts scenes of humanoid figures apparently hunting Sulawesi hairy pigs and anoa [27, 28].

**Fig. 2:**
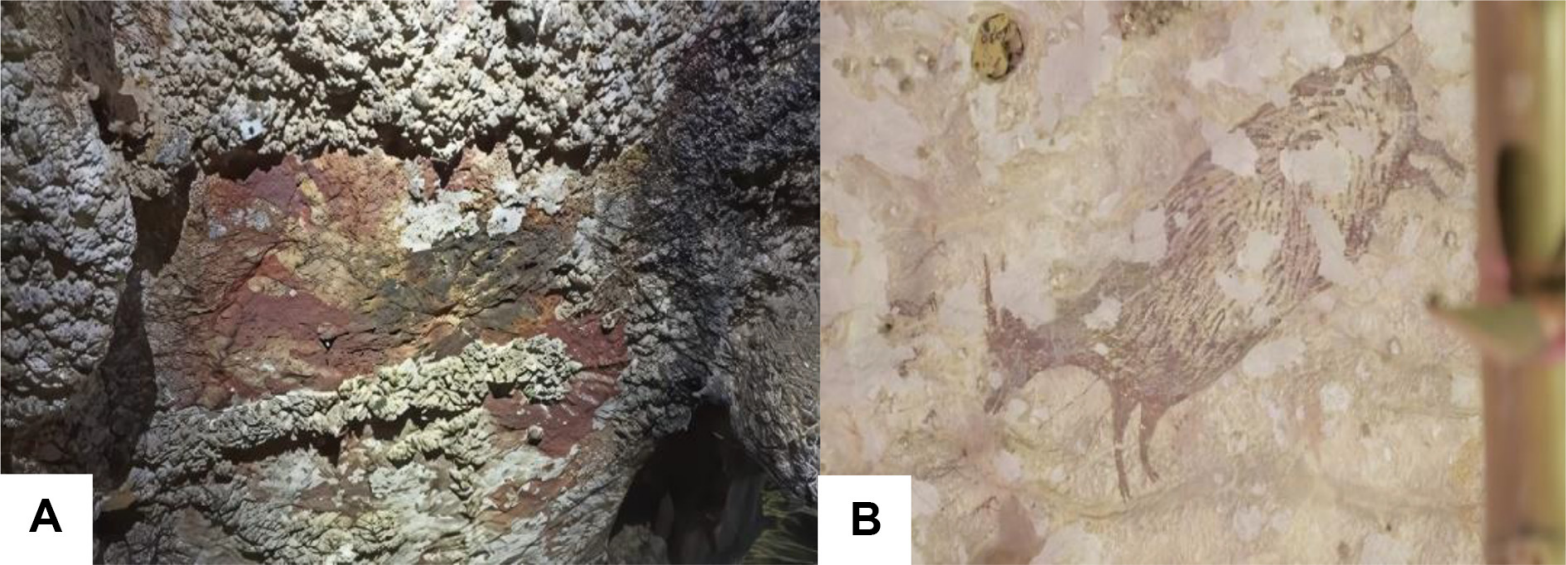
Deposition and deterioration of the paintings: (**A**) Hand stencils in Leang Parewe are beginning to be covered by deposits. (**B**) The painting of a babirusa has suffered significant deterioration.

Most of the paintings at both sites exhibit signs of deterioration, including peeling and partial coverage by deposits of CaCO_3_, rendering some of them incomplete or difficult to discern. In the handprint paintings, sections have either peeled away or are obscured by deposits, resulting in incomplete or indistinct impressions (Fig. 2A). The anoa painting also shows evidence of peeling (Fig. 2B). Furthermore, one painting remains unidentified due to its poor condition. These deposits, which obscure parts of the artwork, are likely formed by microorganisms present in the vicinity of the paintings.

### Sample Collection

Samples were collected from the Maros-Pangkep karst region, specifically from the Parewe and Bulu Sipong caves (Fig. S1). Sampling involved swabbing the surfaces of the karst rock with sterile cotton swabs (Fig. S2). The swabs were then placed in a physiological NaCl solution and stored in a cooling box for transportation. Isolation and identification of the microorganisms were subsequently performed at the Microbiology Laboratory, Department of Biology, Faculty of Mathematics and Natural Sciences, Hasanuddin University, Makassar, Indonesia.

### Isolation and Screening of Carbonatogenic Bacteria

The samples were introduced into 9 ml of physiological NaCl solution and serially diluted up to a 10^-3^ dilution. Each dilution was inoculated onto nutrient agar (NA) medium and incubated at 37°C for 24 h to observe colony formation. Colonies with distinct morphological characteristics were subsequently purified on NA medium until pure isolates were obtained. Screening for carbonatogenic bacteria was performed by inoculating each isolate onto CaCO_3_ precipitation (CCP) medium agar using the quadrant streak method, followed by incubation for 72 h. The presence of carbonatogenic bacteria was indicated by the formation of crystals around the colonies, observed under a stereo microscope.

### Characterization of Carbonatogenic Bacteria

The characterization of carbonatogenic bacteria involved observing bacterial colony morphology, cell morphology, endospore formation, and performing biochemical tests. The biochemical tests included the Sulfide Indole Motility (SIM) test using SIM medium, the citrate test using Simmons Citrate Agar (SCA), the MR-VP test using MR-VP medium, and the catalase test using hydrogen peroxide (H_2_O_2_) [29-33].

### CaCO_3_ Precipitation by Carbonatogenic Bacteria

The precipitation test was conducted to evaluate the bacteria's ability to produce CaCO_3_. A 3 ml bacterial suspension (25% T) was inoculated into 100 ml of NB U/Ca medium and incubated on a shaker (150 rpm) at 30°C for 21 days. This test involved measuring the mass of the formed CaCO_3_ precipitate, analyzing NH_3_ production, and determining the total bacterial count. The presence of CaCO_3_ crystals was indicated by white granules at the bottom of the medium. The CaCO_3_ precipitate was collected by filtration using Whatman No. 42 filter paper and dried in an oven until a constant weight was achieved. The mass of the CaCO_3_ precipitate was then calculated using the formula provided by [34],

Wc=Wfc−Wf (1)

where Wc = precipitant weight (g), Wfc = weight of filter paper containing precipitant (g), and Wf = weight of empty filter paper (g).

### NH_3_ Quantification

NH_3_, a byproduct of urease enzyme activity in bacteria, was measured following filtration of the bacterial cultures using filter paper. A 1 ml sample of the filtrate was transferred to a volumetric flask and diluted to 50 ml with distilled water. From this, 5 ml was transferred into a reaction tube, and 0.5 ml of both Na-phenol and NaOCl solutions were added. The mixture was vortexed until homogeneous and then left undisturbed for 5 min. NH_3_ levels were quantified using a UV-Vis spectrophotometer at a wavelength of 640 nm.

### Total Bacterial Count

Total bacterial counts were performed at 7-day intervals throughout the incubation period. For each time point, the bacterial culture was serially diluted with distilled water and plated onto NA medium. The plates were then incubated for 24 h at 37°C. After incubation, the number of colonies was counted, and the results were used to calculate the total bacterial count using the standard plate count (SPC) formula.

### Molecular Identification

This method involved genomic DNA extraction using the Geneaid Presto™ Mini gDNA Bacteria Kit, followed by DNA amplification through PCR targeting the 16S rRNA gene and utilizing the following primers: Forward 63F (5'-CAGGCCTAACACATGCAAGTC-3') and Reverse 1387R (5'-GGGCGGTGTGTACAAGGC-3'). The PCR products were then separated via electrophoresis on a 1.5% agarose gel. The electrophoresis results were visualized under UV light to detect DNA bands. Samples yielding positive results were subsequently sequenced, and the obtained sequences were analyzed using BLAST (www.ncbi.nlm.nih.gov) for nucleotide identification and homology analysis. This approach enables bacterial identification based on DNA sequences. A phylogenetic tree was constructed using the neighbor-joining method based on the maximum composite likelihood model with evolutionary distance data, as implemented in MEGA11 software [35]. Confidence levels for the phylogenetic tree were assessed through bootstrap analysis with 1,000 replicates.

### Data Analysis

The data for CaCO_3_ precipitate weight, NH_3_ concentration, and total bacterial count were presented in a table. Additionally, the identification results of bacterial isolates obtained through 16S rRNA gene sequencing were visualized in a phylogenetic tree to illustrate the evolutionary relationships, using MEGA-X software.

## Results and Discussion

### Study Area

The sampling sites for carbonogenic bacterial isolates responsible for prehistoric painting damage in this study are in the Pangkajene and Islands Regency, specifically at the two key locations of Leang Parewe and Leang Bulu Sipong 4. Leang Parewe is in Borimasunggu, Labakkang Subdistrict, at coordinates 4°47'40.1''S, 119°31'7.25''E. Leang Bulu Sipong 4 is in Bontoa, Minasatene Subdistrict, at coordinates 4°48'23.81''S, 119°36'36.9''E (Fig. 1A and 1B). Both cave sites are rarely visited and therefore are preserved in relatively pristine conditions.

An examination of the diversity of the prehistoric paintings at the two sites reveals that Leang Parewe contains only handprint paintings, while Leang Bulu Sipong 4 features a wider variety of art, including handprints, footprints, human figures, dogs, anoa (dwarf buffalo), and babirusa (deer-pigs). The oldest images are found on a 4.5-m limestone rock art panel in Leang Bulu Sipong 4, which depicts scenes of humanoid figures apparently hunting Sulawesi hairy pigs and anoa [27, 28].

Most of the paintings at both sites exhibit signs of deterioration, including peeling and partial coverage by deposits (CaCO_3_), rendering some of them incomplete or difficult to discern. In the handprint paintings, sections have either peeled away or are obscured by deposits, resulting in incomplete or indistinct impressions (Fig. 2A). The anoa painting also shows evidence of peeling (Fig. 2B). Furthermore, one painting remains unidentified due to its poor condition. These deposits, which obscure parts of the artwork, are likely formed by microorganisms present in the vicinity of the paintings.

### Isolation of Carbonatogenic Bacteria

Carbonotogenic bacteria were isolated from swab samples taken from prehistoric paintings in the Leang Parewe and Leang Bulu Sipong caves. A total of 18 bacterial isolates with distinct morphological characteristics were obtained: 10 isolates from Leang Parewe, coded as (P), and 8 isolates from Leang Bulu Sipong, coded as (BS). These isolates were then purified to ensure the purity of the bacterial cultures. The presence of bacteria in these cave environments aligns with findings from previous research by [16], who identified 3,562 bacterial isolates belonging to 329 species across 102 genera in two karst areas in Guizhou Province, China.

Macroscopic observations of the isolates revealed that 15 of them displayed irregular shapes and ivory coloring. These isolates were labeled as Ps1-c, Ps1-d, Ps8-a, Ps8-b, Ps8-c, Ps8-d, Ps8-e, BSs2-a, BSs2-c, BSs2-d, BSs2-e, BSs5-a, BSs5-b, BSs5-c, and BSs5-d. In addition, two isolates with circular shapes and milky-white coloring were identified as Ps1-b and Pb1-c, with Ps1-b exhibiting a circular shape and white color.

### Screening of Carbonatogenic Bacteria

The 18 bacterial isolates obtained from the initial isolation were further screened for their ability to precipitate CaCO_3_ using CCP Agar. The screening results revealed 10 positive isolates capable of carbonatogenesis, which were classified as carbonatogenic bacteria (Fig. S4). Six of these isolates originated from Leang Parewe and were identified as Ps1-c, Ps1-d, Ps8-a, Ps8-b, Ps8-c, and Ps8-d. The remaining four isolates, BSs2-a, BSs2-c, BSs2-d, and BSs2-e were from Leang Bulu Sipong. In contrast, the other eight isolates did not demonstrate any crystal formation around their colonies.

The growth of carbonatogenic bacterial isolates was evidenced by the presence of precipitates, specifically CaCO_3_ crystals forming around the bacterial colonies. These findings are consistent with a previous study conducted by [25], where 24 bacterial isolates (23.07% of the total isolates) from Laguna Salada in the Atacama Desert exhibited the ability to produce CaCO_3_ crystals around their colonies, demonstrating a similar capacity for CCP.

### Characterization of Carbonatogenic Bacteria

Morphological characterization of the carbonatogenic bacterial isolates revealed uniformity in colony appearance. All isolates exhibited irregular colony shapes, raised elevations, lobate edges, ivory-white coloration, and a milky-white coloration (Table 1). In terms of cell morphology, six isolates (Ps1-c, Ps1-d, BSs2-a, BSs2-c, BSs2-d, and BSs2-e) were identified as gram-negative Bacilli, while the remaining four isolates (Ps8-a, Ps8-b, Ps8-c, and Ps8-d) were gram-positive Bacilli (Table S1).

Endospore staining confirmed that all isolates could produce spores, thick-walled structures that allow bacteria to withstand extreme environmental conditions. All isolates exhibited positive motility but were negative for hydrogen sulfide (H2S) production and indole formation, indicating the absence of these metabolic pathways. In the citrate test, none of the isolates were able to use citrate as a carbon source. Results from the MR-VP tests showed that all isolates produced mixed acids, as indicated by positive methyl red (MR) results, while only two isolates—Ps8-c and Ps8-d—tested positive for acetoin production in the Voges-Proskauer (VP) test. Catalase tests revealed that all isolates produced catalase (Table 2). In summary, the characterization of carbonatogenic bacteria in this study demonstrated consistent colony morphology across isolates, variations in Gram staining properties, spore-forming abilities, and diverse metabolic profiles, as reflected in their biochemical test results. These traits provide insights into the ecological adaptations and metabolic capacities of these bacteria.

### CaCO_3_ Precipitation by Carbonatogenic Bacteria

**Fig. 3:**
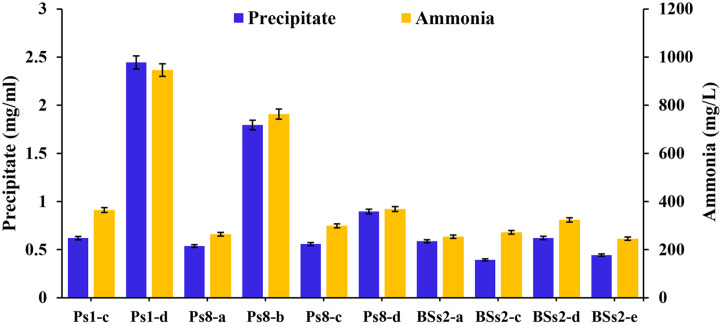
CaCO_3_ and NH_3_ production of carbonatogenic bacteria. Values represent as mean ± standard deviation.

The results showed that all bacterial isolates demonstrated the ability to produce CaCO_3_ with varying concentrations (Fig. 3). The highest precipitate weight was recorded for bacterial isolates Ps1-d at 2.45 ± 0.07 mg/ml and Ps8-b at 1.80 ± 0.05 mg/ml, while the lowest precipitate weight was observed for bacterial isolate BSs2-c, amounting to 0.39 ± 0.01 mg/ml. These findings exceed those of [36], where the highest precipitation value from carbonate-producing bacteria in Riau, Indonesia, was 1.29 mg/ml for isolate sp.20. However, the results are lower than those reported by [37] where isolate LTP4-d from the Maros-Pangkep caves achieved 37.61 ± 0.12 mg/ml. Similarly, [38, 39] reported higher values of 18.2 mg/ml and 16.2 mg/ml, respectively, from *BTPA* 15 and *Lactobacillus macroides* JB2. The variations in precipitate weight are attributed to the distinct urease enzyme-producing capabilities of each bacterium, which influence the amount of CaCO_3_ formed [40]. Other factors influencing bacterial capabilities are the structure and genetic composition of carbonatogenic bacteria, as these play a crucial role in the generation of biomineral composite precipitates [41].

The findings from this study highlight the dual impact of bacterial CaCO_3_ production, particularly in the Maros-Pangkep region, where bacterial activity may contribute to the formation of CaCO_3_ layers that cover ancient rock paintings, potentially leading to the degradation and eventual loss of invaluable cultural heritage. While this calcification poses a threat to delicate artwork, on the other hand, MICP has demonstrated significant benefits in enhancing the mechanical properties of construction materials. Studies have shown that bacterial calcite can effectively fill cracks in concrete, improving durability and extending the lifespan of infrastructure [42, 43]. The efficiency and morphology of CaCO_3_ production are influenced by several factors, including the concentration of calcium ions (Ca^2+^), the availability of dissolved inorganic carbon (DIC), pH levels, and the presence of nucleation sites [44, 45]. Higher Ca^2+^ concentrations and optimal pH can accelerate CaCO_3_ precipitation, while the extracellular polymeric substances (EPS) produced by bacteria can serve as nucleation sites, promoting crystallization [37, 46, 47]. Environmental factors such as temperature, salinity, and the presence of additional ions like zinc also influence both the mineralization process and the crystal morphology [48, 49], further affecting the practical applications of bacterial calcite in various fields. Carbonatogenic bacteria produce carbonate ions by secreting the urease enzyme, which catalyzes the breakdown of urea into NH_3_ and carbonate ions. As this reaction occurs outside the bacterial cell, urease is classified as an extracellular enzyme, enabling it to directly interact with the surrounding environment [50-52].

### NH_3_ Production

The ten bacterial isolates exhibited varying NH_3_ production levels (Fig. 3). The highest NH_3_ concentration was produced by isolate Ps1-d at 946.3 ± 26.3 mg/l, followed by Ps8-b at 763.4 ± 21.2 mg/l, while the lowest NH_3_ concentration was recorded for isolate BSs2-a at 253.7 ± 7.1 mg/l. The NH_3_ levels observed in this study were greater than those found in the earlier work by Zhafirah *et al*. (2024), who recorded 884.72 ± 1.04 mg/l using the bacterium LTP4-d from the karst region of Maros Pangkep [37]. A Pearson correlation analysis revealed a strong positive correlation (*r* = 0.99, *p* = 0.000) between NH_3_ production and CaCO_3_ precipitation. This high correlation suggests that the ureolytic metabolic pathway is the primary mechanism driving MICP by these isolated bacteria (Fig. S5).

NH_3_ is a key product of urea hydrolysis, catalyzed by the urease enzyme [37, 53]. According to Disi *et al*. (2017), bacterial isolates with lower urease activity take longer to hydrolyze urea in the medium, resulting in reduced NH_3_ production [54]. Conversely, isolates with higher urease activity generate more NH_3_, which correlates with increased CaCO_3_ precipitation. This process begins with urea hydrolysis, where urea (CO(NH_2_)_2_) is converted into carbamate (NH_2_COOH), which spontaneously hydrolyzes to form NH_3_ and carbonic acid (H_2_CO_3_). These products further react with water, resulting in the formation of carbonate ions (CO_3_^2-^), ammonium ions (NH_4_^+^), and hydroxyl ions (OH^-^). This series of reactions increases the local pH, creating an alkaline environment. In this alkaline condition, calcium ions (Ca^2+^) present in the medium combine with carbonate ions, and the bacterial cells serve as nucleation sites for CaCO_3_ precipitation.

### Total Bacteria Calculation

Based on the results of the total bacteria calculation (Table 3), we found that the bacterial isolate exhibiting the best growth was the Ps1-d isolate. The variation in total bacteria increase is attributed to differences in the quality of urease enzymes produced. The bacteria-produced urease enzyme is utilized to break down urea present in the medium. The added urea serves as a nitrogen source for carbonatogenic bacteria. Bacteria require a nitrogen source to support their maximal growth as nitrogen is a key component in the formation of proteins, enzymes, and nucleic acids [55].

### Molecular Identification of Carbonatogenic Bacteria

Molecular analysis of the 16S rRNA gene was performed on the two bacterial isolates exhibiting the highest NH_3_ production and CaCO_3_ precipitation, namely Ps1-d and Ps8-b. Sequencing results, analyzed using the BLAST program against the 16S ribosomal RNA sequence database (Table 4), revealed that isolate Ps1-d showed the highest similarity to *Bacillus cereus* strain bk, with a sequence similarity of 97.59% and an error value of 0.0, based on alignment with GenBank data. Similarly, isolate Ps8-b was most closely related to *Bacillus* sp. NCCP-428, with a sequence similarity of 98.37% and an error value of 0.0.

**Fig. 4:**
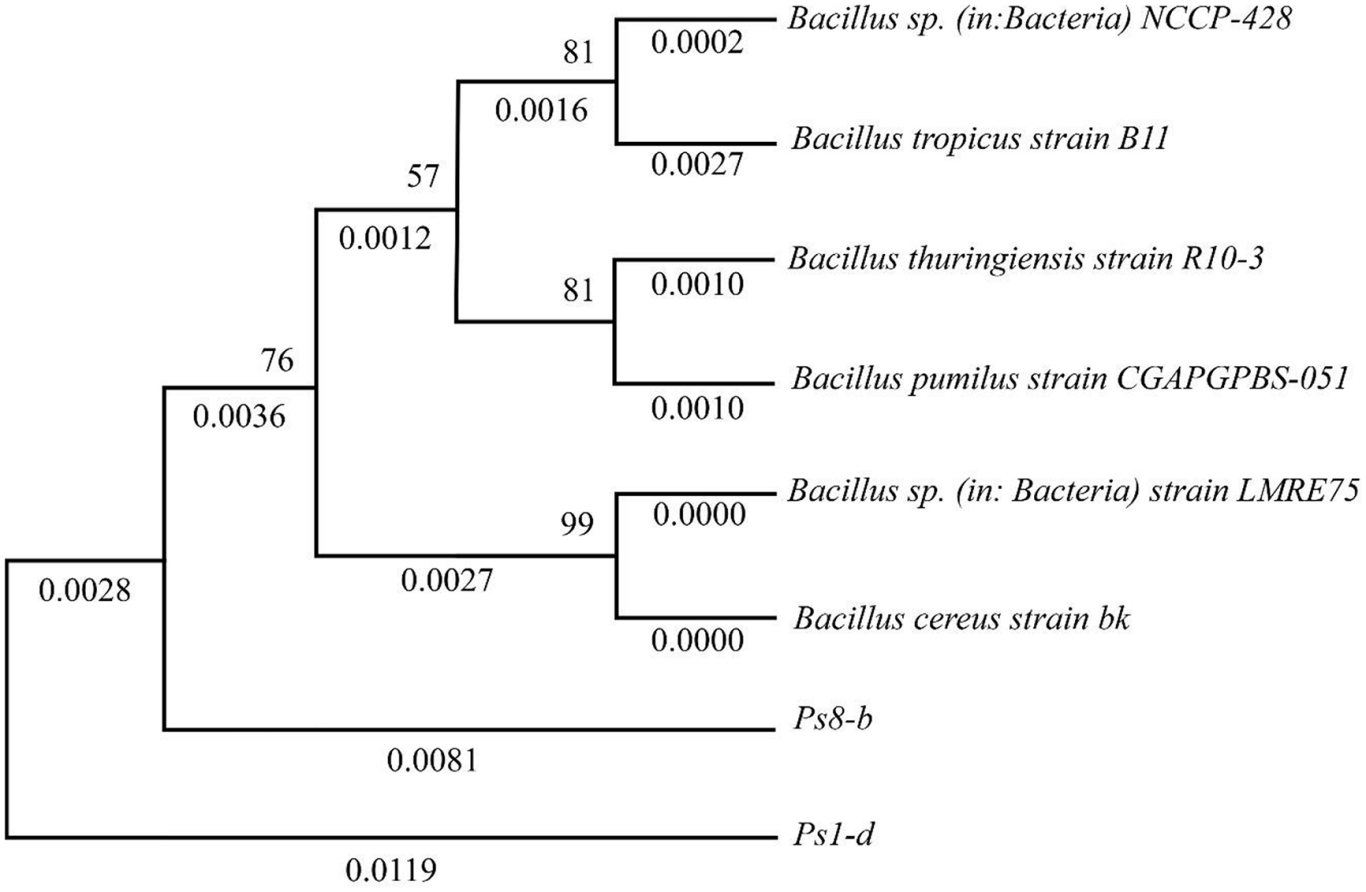
Phylogenetic tree with UPGMA method for Ps1-d and Ps8-b isolates. The tree shows the evolutionary relationships of isolates with reference *Bacillus* species. Branch values indicate genetic distances, and numbers at the nodes represent bootstrap support.

The sequencing results of bacterial isolates Ps1-d and Ps8-b, analyzed using the BLAST NCBI program, were further employed to construct a phylogenetic tree using the UPGMA method in the MEGA X software, as shown in Fig. 4. The isolate Ps1-d demonstrates an almost complete sequence identity with *Bacillus cereus* strain bk, exhibiting a genetic distance of 0.0000, which strongly suggests that Ps1-d is a strain of *B. cereus*. This phylogenetic relationship is further supported by a high bootstrap value of 99, indicating strong confidence in the robustness of the clustering. In contrast, the Ps8-b isolate is closely related to *Bacillus* sp. NCCP-428, with a genetic distance of 0.0028, indicating a high degree of similarity, though not as identical as Ps1-d to *B. cereus*. The bootstrap value of 76, while lower, still provides moderate support for this relationship, suggesting that Ps8-b is likely a closely related strain within the B. genus.

These findings align with previous research conducted in two unexploited karst caves in the Kuankuoshui Nature Reserve, Guizhou Province, China, where 8.8% of the identified bacterial population was classified under the genus *Bacillus* based on 16S rRNA gene analysis [16]. Similarly, in the study "Bioprospecting of Ureolytic Bacteria from Laguna Salada," ureolytic bacteria from the genera *Bacillus*, *Salinivibrio*, *Halomonas*, *Pseudomonas*, and *Porphyrobacter* were identified, showing sequence similarities ranging from 98% to 100% based on 16S rRNA sequences analyzed via BLAST [25]. Like the current study, these findings underscore the widespread presence of *Bacillus* species in ureolytic bacterial communities, further emphasizing their critical role in CaCO_3_ precipitation through the MICP pathway.

The potential applications of carbonatogenic bacteria in cultural heritage conservation are twofold. First, these bacteria could be harnessed for restoration and conservation efforts across a variety of heritage materials, particularly in stone artworks. Their ability to induce biomineralization, specifically the formation of CaCO_3_, offers a natural, efficient solution for reinforcing degraded substrates. This method provides an alternative to synthetic conservation materials, such as acrylic and epoxy resins, copolymers, alkoxysilanes, and phosphates, which often exhibit several drawbacks, including chemical-physical incompatibility, insufficient penetration, and high degradation rates—especially in organic compounds. By contrast, bacterial biomineralization could form a durable and coherent protective layer, helping to consolidate stone surfaces while mitigating the negative effects of weathering and other environmental stresses. This approach offers eco-friendly conservation solutions. Applied via sprays or gels, it complements traditional methods like protective coatings while minimizing risks through strain selection and environmental control [56].

Second, these bacteria could serve as a model for studying how to inhibit unwanted CaCO_3_ precipitation in cases where surface deposition might lead to degradation, such as in painted wall art. Understanding the conditions under which these bacteria induce or inhibit calcite precipitation could inform strategies to prevent further damage to valuable paintings. This insight would be particularly relevant to mitigating the risk of deterioration caused by the excessive deposition of CaCO_3_ on surfaces that should remain free from such mineral layers, such as prehistoric artworks. The dual potential of carbonatogenic bacteria, both as a conservation agent and as a model for inhibiting deleterious calcite precipitation, could revolutionize current practices in the preservation of cultural heritage. Further research should focus on optimizing bacterial applications for different substrates and environments, ensuring that they can be tailored for specific conservation needs while minimizing the risk of unintended damage.

This research highlights the importance of refining application techniques to ensure these processes are effectively harnessed while minimizing unintended consequences. Equally important is the development of inhibition strategies for scenarios where CaCO_3_ formation could harm delicate artworks, such as prehistoric cave paintings. This might involve exploring inhibitors that selectively suppress bacterial urease activity or calcium ion availability, effectively halting excessive calcification without disrupting other environmental factors. Additionally, environmental modulation, such as regulating humidity, temperature, and pH, can either enhance or inhibit bacterial activity based on conservation needs [57, 58]. Bacterial calcification can be integrated with advanced protective coatings, such as those based on acrylic polymers (*e.g.*, polyacrylates and methacrylates), which have a long history of use in heritage preservation [59, 60]. Coatings infused with bioactive agents could potentially allow simultaneous protection and controlled calcification to address the needs of vulnerable substrates like cave paintings. Inhibitor-enhanced coatings may also provide selective protection in cases where environmental factors are difficult to control.

While this study demonstrates the potential of carbonatogenic bacteria in precipitating CaCO_3_, their application in real-world conservation settings is subject to several environmental limitations that warrant further investigation. Factors such as temperature, humidity, and the composition of the surrounding environment can significantly impact the effectiveness of bacterial calcification. For instance, moderate increases in temperature within the physiological limits of the bacteria can enhance urease activity, thereby accelerating urea hydrolysis and carbonate ion production [47, 61]. However, extreme temperatures may denature essential enzymes or inhibit bacterial growth, limiting their viability in certain climates. Similarly, fluctuations in humidity can influence the availability of moisture necessary for bacterial activity and CaCO_3_ precipitation, while the presence of competing ions or other minerals in the environment may disrupt or modify the precipitation process.

## Conclusion

The isolation of bacteria from Maros-Pangkep limestone rocks resulted in 18 isolates, with 10 from Parewe Cave and 8 from Bulu Sipong Cave. Among these, 10 isolates were identified as carbonatogenic bacteria. Quantitative testing of CaCO_3_ precipitation revealed that isolates Ps1-d and Ps8-b exhibited the highest precipitatation of CaCO_3_ at 2.45 ± 0.07 mg/ml and 1.80 ± 0.05 mg/ml, respectively. The highest NH_3_ concentration was also produced by isolate Ps1-d at 946.3 ± 26.3 mg/l and Ps8-b at 763.4 ± 21.2 mg/l. Molecular identification classified Ps1-d as *Bacillus cereus* strain bk and Ps8-b as *Bacillus* sp. NCCP-428. These findings highlight the significant roles of carbonatogenic bacteria in cultural heritage conservation. Their biomineralization capabilities could be utilized to restore and reinforce stone artworks by creating durable, natural protective layers, offering a sustainable alternative to synthetic materials. Furthermore, understanding how these bacteria induce or inhibit CaCO_3_ precipitation may inform strategies to prevent unwanted mineral deposition on delicate surfaces, such as wall paintings. Future research should focus on optimizing these applications as safe conservation solutions.

## Figures and Tables

**Table 1:** Characterization of carbonatogenic bacteria.

Isolate	Colony Morphology				Cell Morphology		
	Shape	Elevation	Edge	Color	Shape	Gram	Endospore
Ps1-c	Irregular	Raised	Lobate	Ivory white	Bacil	Negative	+
Ps1-d	Irregular	Raised	Lobate	Milky white	Bacil	Negative	+
Ps8-a	Irregular	Raised	Lobate	Ivory white	Bacil	Positive	+
Ps8-b	Irregular	Raised	Lobate	Ivory white	Bacil	Positive	+
Ps8-c	Irregular	Raised	Lobate	Ivory white	Bacil	Positive	+
Ps8-d	Irregular	Raised	Lobate	Ivory white	Bacil	Positive	+
BSs2-a	Irregular	Raised	Lobate	Ivory white	Bacil	Negative	+
BSs2-c	Irregular	Raised	Lobate	Ivory white	Bacil	Negative	+
BSs2-d	Irregular	Raised	Lobate	Ivory white	Bacil	Negative	+
BSs2-e	Irregular	Raised	Lobate	Ivory white	Bacil	Negative	+

*+: Endospore detected; -: Endospore undetected

**Table 2:** Biochemical testing of carbonatogenic bacteria.

Isolate	SIM			Citrate	MR-VP		Catalase
	Motility	H_2_S	Indole		MR	VP	
Ps1-c	Motile	-	-	-	+	+	+
Ps1-d	Motile	-	-	-	+	+	+
Ps8-a	Motile	-	-	-	+	-	+
Ps8-b	Motile	-	-	-	+	-	+
Ps8-c	Motile	-	-	-	+	-	+
Ps8-d	Motile	-	-	-	+	-	+
BSs2-a	Motile	-	-	-	+	-	+
BSs2-c	Motile	-	-	-	+	-	+
BSs2-d	Motile	-	-	-	+	-	+
BSs2-e	Motile	-	-	-	+	-	+

*+: Positive to produce; -: Negative to produce

**Table 3:** CaCO_3_ and NH_3_ production of carbonatogenic bacteria.

Isolate	Total Bacteria (Log CFU/ml)			
	T0	T7	T14	T21
Ps1-c	6.7	12.3	15.1	15.0
Ps1-d	6.7	15.9	16.8	16.8
Ps8-a	7.1	12.8	16.5	16.9
Ps8-b	6.7	12.7	14.7	14.3
Ps8-c	6.3	11.6	13.5	15.5
Ps8-d	6.6	13.9	16.8	16.2
BSs2-a	6.9	11.5	15.0	15.0
BSs2-c	6.7	12.2	12.7	13.1
BSs2-d	6.9	12.3	14.9	16.9
BSs2-e	6.9	10.8	13.7	13.7

*Data represented as mean ± SD; T0: Day 0; T7: Day 7; T14: Day 14; T21: Day 21

**Table 4:** BLAST Results of PS1-D and PS8-B.

Isolate	Species	Query Cover	E Value	Per. Ident
Ps1-d	*Bacillus cereus* strain bk	91%	0,0	97,59%
Ps8-b	*Bacillus* sp. (in: Bacteria) NCCP-428	99%	0,0	98,37%
